# Favorable response of a patient with primary B/myeloid mixed phenotype acute Leukemia to CD19-CAR-T: Case report and literature review

**DOI:** 10.1097/MD.0000000000036397

**Published:** 2023-12-15

**Authors:** Lixin Wang, Yanbin Pang, Chuling Fang, Weiqiang Zhao, Yuanyuan Xu, Xiao Guo, Jingqiao Qiao, Junhui Mei, Hongxin Wang, Chuan Yu, Yisheng Li, Zhixiong Tang, Li Yu

**Affiliations:** a Department of Hematology and Oncology, Shenzhen University General Hospital, International Cancer Center, Shenzhen Key Laboratory, Hematology Institution of Shenzhen University, Shenzhen University Health Science Center, Shenzhen University, Shenzhen, China; b Shenzhen Haoshi Biotechnology Co., Ltd, Shenzhen, China; c Shenzhen University – Haoshi Cell Therapy Institute, Shenzhen, China; d Shenzhen Hospital of Southern Medical University/Shenzhen Clinical Medical school, Shenzhen, China.

**Keywords:** case report, CD19-CAR-T, MPAL

## Abstract

**Rationale:**

Mixed phenotype acute leukemia (MPAL) is a rare and heterogeneous type of leukemia known for its poor prognosis. The optimal treatment strategy for this condition currently lacks consensus, leaving uncertainty in its management. Nonetheless, a potential therapeutic option for patients with refractory MPAL who express target antigens is donor-derived chimeric antigen receptor T (CAR-T) cell therapy.

**Patient concerns:**

We recently reported a 61-year-old woman with MPAL and elucidated its diagnosis and treatment.

**Diagnosis:**

The diagnosis of MPAL was established based on the classification of World Health Organization in 2016.

**Interventions:**

Despite undergoing 3 different acute lymphoblastic leukemia (ALL) regimens and 1 acute myelogenous leukemia (AML) regimen, the patient did not achieve remission. Subsequently, the patient received human CD19-targeted CAR-T cell therapy.

**Outcomes:**

The patient achieved a successful and complete remission after CAR-T cell therapy. Tragically, 8 months after CAR-T infusion, the patient experienced a relapse characterized by CD19-negative disease and ultimately passed away.

**Lessons:**

This case underscores the potential efficacy and safety of human-derived CD19 CAR-T cell therapy in treating refractory MPAL. While this particular patient outcome was unfortunate, it suggests that CAR-T cell therapy may still hold promise as a viable treatment option for MPAL patients unresponsive to other therapies. Further research in this field is warranted to determine the most effective treatment strategies for managing this challenging disease.

## 1. Introduction

Mixed phenotype acute leukemia (MPAL) is a highly heterogeneous leukemia with exceptionally rare incidence, comprising only 2% to 5% of leukemia. Characterized by co-expression of myeloid and lymphoid markers, MPAL can be classified into various subtypes: B/myeloid, T/myeloid, B/T, and trilineage, with B/myeloid and T/myeloid are the 2-principal subtype.^[1–5]^ MPAL is a high-risk leukemia that carries the worst prognosis among acute leukemias according to the Surveillance, Epidemiology, and End Results dataset.^[6]^ The median survival time of adult patients with MPAL remains <3–6 months. Its optimal treatment strategy remains obscure and no consensus has been reached.^[5]^ Acute lymphoblastic leukemia (ALL) and hybrid regimens are associated with higher initial remission rates than more toxic acute myeloid leukemia (AML) regimens.^[4]^ There are few reports on the efficacy of CAR-T therapy on MPAL.

We presented a case report of a 61-year-old female patient diagnosed with B/myeloid MPAL who underwent 3 different ALL regimens and 1 AML regimen but failed to achieve remission. Following this, the patient received human CD19-targeted CAR-T cell therapy, which led to complete remission.

## 2. Case presentation

The patient visited our hospital for exertional dyspnea for 4 weeks on December 21th, 2021. Physical examination did not reveal any enlarged lymph nodes or splenomegaly. Routine blood test showed leukocytosis (26.46 × 10^9^/L) without anemia or thrombocytopenia. Peripheral blood smear revealed 56.0% of blast cells. Bone marrow examination presented an increase of blasts up to 78.5%, 55% of which stained positively for myeloperoxidase. Flow cytometry analysis of bone marrow showed a group of abnormal blast population (87.57%) that expressed both B-cell and myeloid lineages markers, including CD19, CD34, CD38, HLA-DR, CD33, CD13, cMPO, cCD22dim, cCD79a, with a small part expressed CD10 and CD117. Cytogenetic studies showed a normal karyotype. Molecular analysis failed to detect any fusion gene, including BCR-ABL. While missense mutation of FLT3 (FLT3-ITD: (2039C > T (p.A680V) exon16 (VAF of 20.1%) (703X); 1727 > C (p.L576P) exon14 (VAF of 2.1%) (1168X)) and TET2 (VAF of 42.2%) was identified according to next generation sequencing (NGS). Collectively, the patient was diagnosed with B/myeloid MPAL by WHO classification.

The patient initially received 2 cycles of induction chemotherapy with an ALL-based regimen (cytarabine, dexamethasone, vindesine, daunorubicin and VDCP, respectively). Unfortunately, after each chemotherapy, bone marrow examination was still hyperplasia, and the blasts were 73.5% to 94%, indicating failure of ALL regimens. So the patient received a regimen consisting of cyclophosphamide, vindesine, dexamethasone, etoposide and venetoclax. However, after 20 days of the regimen, bone marrow smear revealed 87% blast cells. Since the NGS showed mutation burden of FLT3 (NM_004119:c.2039C > T exon16 (20.1%) and c.1727TC > C exon14 (2.1%)), gilteritinib (120mg qd po, d1-10), an inhibitor of mutant FLT3, and decitabine (25mg d1-5) were prescribed. Nevertheless, bone marrow smear on day 7 also showed an increase of blast cells (88.0%). Till now, it seemed that the patient hardly benefited from traditional combination chemotherapy. The positive rate of CD19 in blast cells was still as high as 88%. Therefore, CD19-targeted CAR-T cell therapy, a pronouncing treatment for ALL, was conducted.

**Figure 1.**
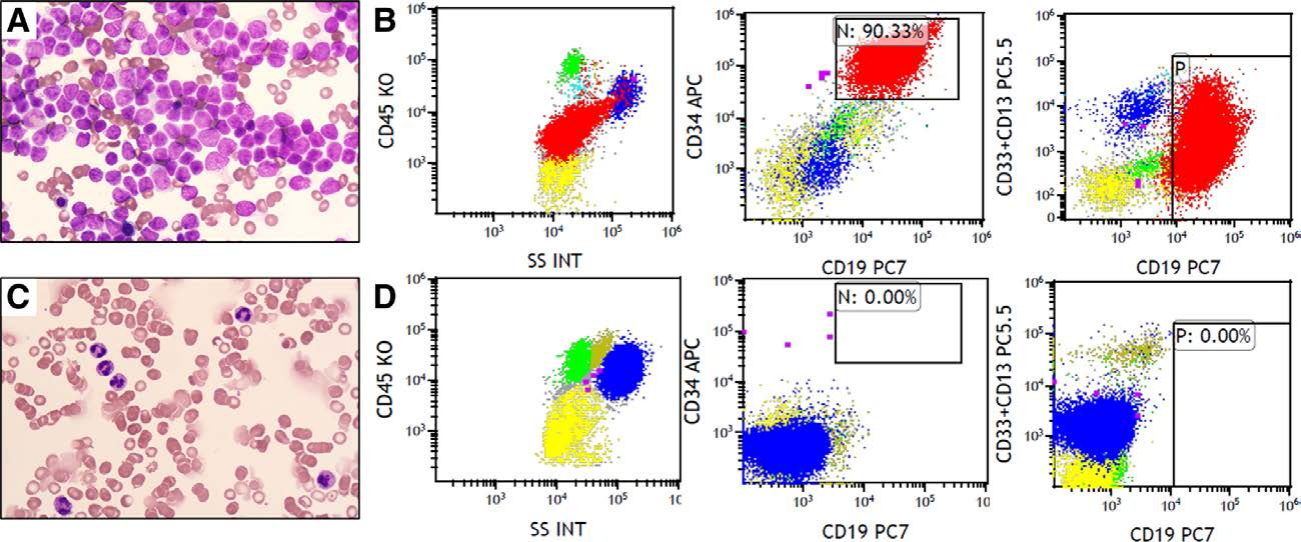
Morphology and immunophenotypic characteristics of bone marrow samples before and after CAR-T treatment. (A) There were a large number of blast cells in the bone marrow before CAR-T cell therapy. (B) Flow cytometry from bone marrow samples showed a large number of B/myeloid biphenotypic blast cells before CAR-T therapy. (C) Morphological remission on day 27 after CAR-T cells therapy; (D) On day 27 after CAR-T therapy, flow cytometry of bone marrow samples showed no B/myeloid biphenotypic blast cells. CAR-T = chimeric antigen receptor T.

Before CAR-T cell infusion, the patient received a standard lymphodepleting regimen based on fludarabine (25 mg/m^2^, day −5 ~ −3) and cyclophosphamide (250mg/m^2^, day −5 ~ −3). Just before cell infusion, bone marrow smear detected 90% of blast cells (Fig. 1A and 1B). Then, 0.37 × 10^6^/kg CD19 CAR-T cells produced by Shenzhen Haoshi Biotechnology Co., Ltd., were infused according to our CAR-T clinical practice guidelines. The total dose was 2.7* × *10^8^, and the positive rate was 8.1%.

**Figure 2.**
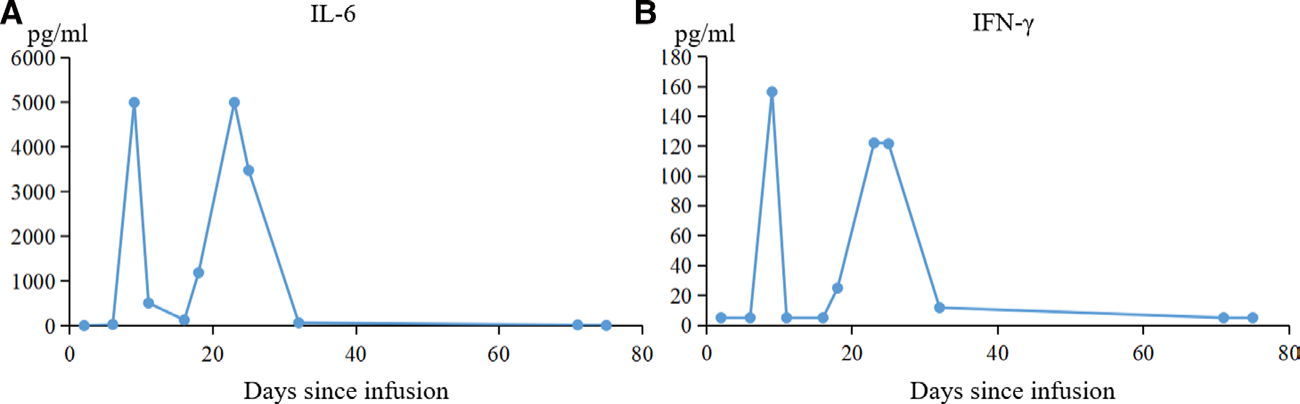
Changes of cytokines during CAR-T treatment. (A, B) Levels of IL-6 and IFN-γ after CAR-T cell therapy, which showed obvious double peaks. CAR-T = chimeric antigen receptor T.

**Figure 3.**
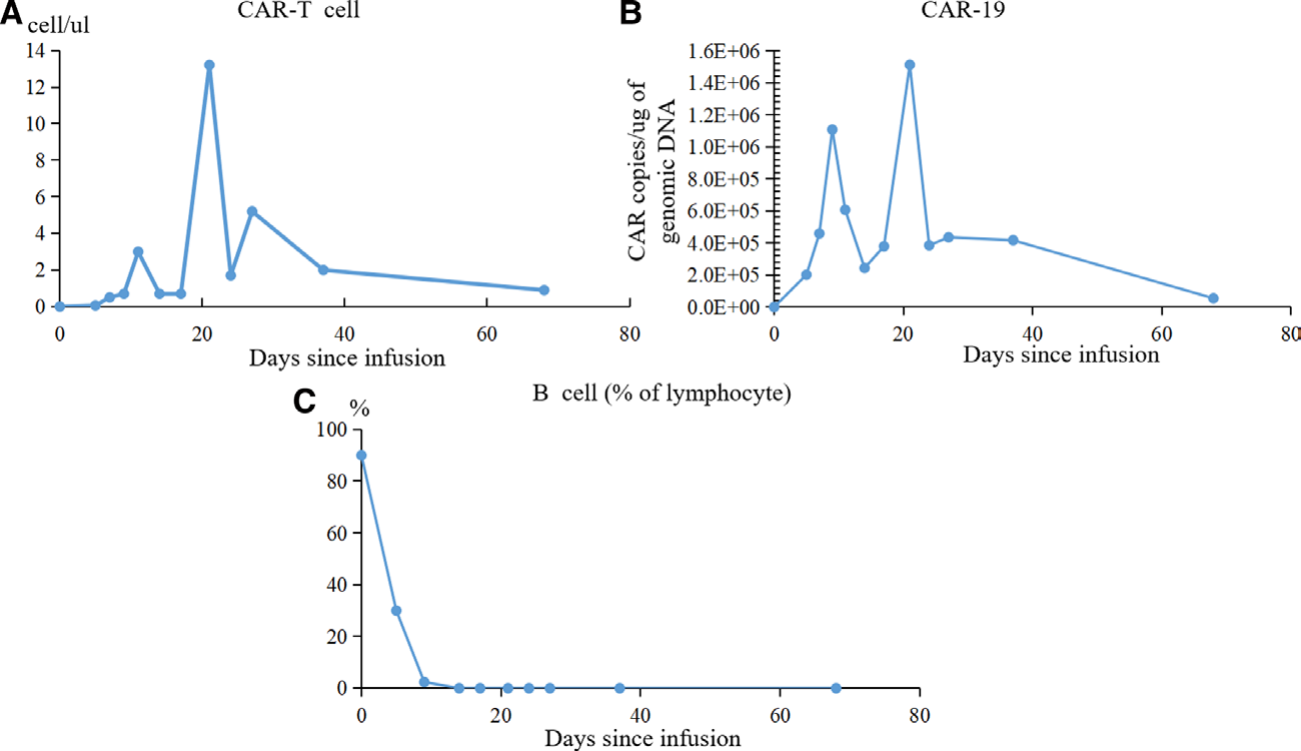
Response to CAR-T cell therapy. (A, B) Number of CAR T cells and lentivirus-containing CAR copies in the peripheral blood after CAR-T cell therapy. The expansion of CAR-T cells showed obvious double peaks. (C) Changes of B cell during CAR-T cell therapy. CAR-T = chimeric antigen receptor T.

On day 8 after the infusion, the patient developed a persistent high temperature for 4 days, with a maximum temperature of 40.0°C. There were no hypoxia, tachycardia, epilepsy, hypotension, or life-threatening events, such as multiorgan dysfunction after transfusion or disseminated intravascular coagulation. Peak levels of the IL-6 and IFN-γ in the patient blood were observed before each of 2 fast CAR-T cells expansion periods around day 9 and day 21 (Fig. 2A and 2B). On day 17, the patient developed grade I CRS, which was well managed with supportive care. The dynamic expansion of CAR-T cells in peripheral blood was monitored by flow cytometry (FC) and quantitative polymerase chain reaction (qPCR). The absolute CAR-T cell counts peaked on day 21, consistent with the CAR copy number in peripheral blood (Fig. 3A and 3B), while no residual CD19-positive B lymphocyte was detected after 14 days by flow cytometry (Fig. 3C). Bone marrow examination showed hypoplasia without blast cells on day 27 (Fig. 1C). Meanwhile, FC showed an MRD-negative status on day 27 (Fig. 1D). The RBT showed WBC 6.15 × 10^9^/L, lymphocyte percentage 10.9%, hemoglobin level 79g/L, and platelet count 124 × 10^9^/L on day 26. Finally, complete remission was achieved. Considering the predictable poor prognosis, hematopoietic stem cell transplantation should have been recommended, however, this patient rejected treatment and signed for discharge. Unfortunately, 2 months after the infusion of CD19 CAR-T cells, the patient experienced a relapse, with 20.5% blast cells detected in their bone marrow via flow cytometry. Notably, these blast cells did not express CD19. Unfortunately, the patient died 8 months after CAR-T infusion due to the disease relapse.

All procedures performed in this study adhered to the ethical standards of the institutional and/or national research committee(s) and the Helsinki Declaration. Written informed consent was obtained from the patient for publication of this case report and its associated images.

## 3. Discussion

MPAL is an extremely rare leukemia exhibiting a complicated phenotype. Due to the lack of large-scale cohort studies, treatment strategies for MPAL have been mainly reported through case reports, and no consensus on treatment has been reached so far.^[5]^ While, according to meta-analysis, ALL-regimen was proved to be the most valuable strategy for MPAL.^[5]^ Rasekh et al conducted a retrospective analysis of 102 patients with MPAL who received ALL-like treatment, the results showed the CR rate of children was 77.8%%, while that of adults (age ≥ 18 years) was only 51.5%% (*P* = .015).^[7]^ In our case, the patient failed to respond to the first 2 ALL regimens. In accordance with the results of the aforementioned study of Rasekh which showed that the response of children to ALL therapy was significantly superior to that of adults. This may indicate that ALL-like regimens may not be completely applicable to adult MPAL, especially elderly patients, as they are for ALL.

In our case, since the failure of ALL-based therapy, an AML-regimen consisting of decitabine and gilteritinib (FLT3 inhibitor) was given as a salvage treatment for the patient. However, this regimen had no effect either. The FMS-related tyrosine kinase 3 (FLT3) mutation not only confers a poor prognosis but also increases the risk of failure of initial therapy. Recent studies showed that the FMS-related tyrosine kinase 3 (FLT3) gene mutation was found in 12% to 30% of all MPAL.^[8–10]^ However, due to the rarity of MPAL with FLT3 mutations, the effect of FLT3 mutations on prognosis is uncertain, and there is little evidence to guide the treatment of FLT3-positive MPAL.

CD19 CAR-T has been successfully used in B cell malignancy, and the promising effect of CD19 CAR-T shed a light on the treatment of this patient. Since the patient exhibited a B malignant phenotype and FC showed CD19 (88%) expressed, it was reasonable to hypothesize that patients with B/myeloid would benefit from CD19 CAR-T therapy. Following conventional lymphoid depletion treatment, CD19 CAR-T cell was infused. Finally, complete remission was achieved. However, without bridging hematopoietic stem cell transplantation, the patient eventually morphological relapsed after 1 month of CR.

The outstanding features of the patient during CAR-T treatment are as follows: This case was an elderly patient with primary drug-resistant MPAL accompanied by FLT3 mutation. Despite there have been successful treatment of MPAL with CAR-T or blinatumomab, none of these cases included patients with FLT3 mutation. Patients with FLT3 mutation were also excluded from the clinical trials of CAR-T in the treatment of AML. The success of this case provides a clinic valuable exploration for CAR-T in CD19^+^MPAL with FLT3 mutation. CAR-T combined with FLT3 inhibitors may be a useful therapeutic option for this population. Although the blast cells of the patient were up to 90% before CAR-T treatment, the patient successfully achieved complete remission with high safety. There are several limitations in this case. Firstly, midostaurin was not incorporated into the induction therapy, even though the patient had low-frequency mutations. Adding midostaurin to the treatment regimen of patients with FLT3 mutations could significantly improve the efficacy, which has been confirmed not only in AML but also in MPAL. Secondly, only the quantity of CAR-T cells was measured, with no analysis of their functionality. It remains unclear why such a limited number of CAR-T cells could lead to remission in patients with refractory MPAL.

## 4. Conclusion

Our case demonstrates the efficacy and safety of CD19 CAR T cell infusion in elderly MPAL patients with primary resistance and FLT3 mutations. Even in cases where CAR-T cells do not lead to long-term remission, it is still possible to achieve a morphological remission in elderly MPAL with primary resistance and FLT3 mutation.

## Author contributions

**Conceptualization:** Chuling Fang, Lixin Wang, Yanbin Pang, Weiqiang Zhao, Xiao Guo, Jingqiao Qiao, Hongxin Wang, Chuan Yu, Yisheng Li, Zhixiong Tang, Li Yu.

**Data curation:** Chuling Fang, Lixin Wang, Yanbin Pang, Weiqiang Zhao, Xiao Guo, Jingqiao Qiao, Junhui Mei, Chuan Yu, Yisheng Li, Zhixiong Tang, Li Yu.

**Formal analysis:** Chuling Fang, Yanbin Pang, Yuanyuan Xu, Xiao Guo, Chuan Yu, Zhixiong Tang.

**Funding acquisition:** Lixin Wang, Li Yu.

**Investigation:** Chuling Fang, Lixin Wang, Yanbin Pang, Weiqiang Zhao, Yuanyuan Xu, Xiao Guo, Junhui Mei, Hongxin Wang, Yisheng Li, Li Yu.

**Methodology:** Chuling Fang, Yanbin Pang, Weiqiang Zhao, Yuanyuan Xu, Xiao Guo, Jingqiao Qiao, Junhui Mei, Hongxin Wang, Yisheng Li.

**Project administration:** Lixin Wang, Yuanyuan Xu, Xiao Guo, Junhui Mei, Chuan Yu.

**Resources:** Lixin Wang, Yisheng Li, Zhixiong Tang, Li Yu.

**Supervision:** Chuling Fang, Yuanyuan Xu, Hongxin Wang, Li Yu.

**Software:** Jingqiao Qiao, Hongxin Wang, Chuan Yu, Yisheng Li, Zhixiong Tang.

**Validation:** Yuanyuan Xu, Jingqiao Qiao.

**Visualization:** Chuling Fang, Jingqiao Qiao, Junhui Mei.

**Writing – original draft:** Yanbin Pang.

**Writing – review & editing:** Chuling Fang, Lixin Wang, Yuanyuan Xu, Xiao Guo, Jingqiao Qiao, Junhui Mei, Hongxin Wang, Chuan Yu, Yisheng Li, Zhixiong Tang, Li Yu.
